# Trees as brokers in social networks: Cascades of rights and benefits from a Cultural Keystone Species

**DOI:** 10.1007/s13280-022-01733-z

**Published:** 2022-06-23

**Authors:** Houria Djoudi, Bruno Locatelli, Catherine Pehou, Matthew J. Colloff, Marlène Elias, Denis Gautier, Russell Gorddard, Barbara Vinceti, Mathurin Zida

**Affiliations:** 1 Jalan CIFOR Situ Gede, Bogor Barat, Bogor, 16115 Indonesia; 2 Cirad, UPR Forêts et Sociétés, Univ Montpellier, TA C105-D, 34398 Montpellier Cedex5, France; 3 Center for International Forestry Research (CIFOR), 06 BP 9478 Ouagadougou, Burkina Faso; 4 Fenner School of Environment and Society, Australian National University, Bldg, 141, Linnaeus Way, Canberra, ACT 2601 Australia; 5 The Alliance of Bioversity International and International Center for Tropical Agriculture, Via di San Domenico, 1, 00153 Rome, Italy; 6 CSIRO Land and Water, Ngunnawal Country GPO, Box 1700, Canberra, ACT 2601 Australia

**Keywords:** Adaptation, Agency, Burkina Faso, Cultural Keystone Species, Social–ecological network analysis, Traditional ecological knowledge

## Abstract

Indigenous trees play key roles in West African landscapes, such as the néré tree (*Parkia biglobosa* (Jacq.) R.Br. ex G.Don). We applied social–ecological network analysis to understand the social–ecological interactions around néré. We documented the benefits néré provides and the multiple social interactions it creates amongst a large range of actors. The flows of rights over the trees and benefits from them formed two hierarchical networks, or cascades, with different actors at the top. The two forms of power revealed by the two cascades of rights and benefits suggest possible powers and counter-powers across gender, ethnicity, and age. We documented how the tree catalyses social interactions across diverse groups to sustain vital social connections, and co-constitute places, culture, and relationships. We argue that a paradigm shift is urgently needed to leverage the remarkable untapped potential of indigenous trees and Cultural Keystone Species in current global restoration and climate change agendas.

**Supplementary Information:**

The online version contains supplementary material available at 10.1007/s13280-022-01733-z.

## Introduction

Trees occupy an important part of the savannah mosaic landscapes of West Africa, which include woodlands, pasture, and farmlands interspersed with extensive agroforestry parklands (Raynaut 2001; Hanan 2018). As the backbone of millions of livelihoods, trees play a crucial role in sustaining life in a changing climate (Bayala et al. 2014; Koffi et al. 2020). They are also central to the collective imaginaries and symbolic expression of the different cultures and identities of the inhabitants of those landscapes (Pélissier 1980).

Culturally important trees, such as néré (African locust bean—*Parkia biglobosa* (Jacq.) R. Br. ex G. Don), baobab (*Adansonia digitata* L.) and shea (*Vitellaria paradoxa* C.F. Gaertn.), differ not only in the benefits they provide to people, but also in terms of the complex rights and access arrangements associated with them, which have been fine-tuned over centuries through social relationships and landscape management by diverse actors (Poudyal 2009; Ræbild et al. 2012; Rousseau et al. 2017; Pehou et al. 2020).

Previous research on trees in the West African savannahs has highlighted the role of néré in traditional food systems (Ouoba et al. 2005; Koura et al. 2011; Touré 2018), nutritional security (Vinceti et al. 2018; Koffi et al. 2020; Termote et al. 2020), livelihoods (Kronborg et al. 2013; Pouliot and Treue 2013; Termote et al. 2020), traditional medicine (Arbonnier 2009; Asuzu and Harvey 2003), and access and rights (Gausset et al. 2005; Coulibaly-Lingani et al. 2009; Pehou et al. 2020). However, research is lacking on the centrality of néré in socio-cultural interactions and its role in driving or influencing access rights and benefits within social networks and power relations.

Social relations determine the way people interact with trees and other non-human elements such as land or water (Hageneder 2001; Colloff 2020). This is particularly important for trees that are common resources, because networks and social capital, including sharing and collective practices, shape and build the rules and norms of how the commons are used and maintained (Ostrom 1990). Rather than being static, those interactions are relations-in-the-making, constantly renegotiated and reconfigured (Nightingale 2019).

Social relations and the interactions between people and trees are driven by power relationships. Power imbalances can have adverse outcomes, for example when a particular group benefits from a tree resource whilst denying access to another group (Lybbert et al. 2002; Elias and Carney 2005; Buchmann et al. 2010; Rousseau et al. 2017). The importance of power in social networks is widely accepted, but it is challenging to understand context-specific power dynamics, and identify how inclusions and exclusions are lived and contested. Several theories can be used to interpret different forms of power in social interactions, such as the Resource Dependence Theory (Reimann and Ketchen 2017), the Social Exchange Theory (Blau 1964; Emerson 1976), and the Social Theory of Reciprocity and Gift Exchange (Mauss 2002). The multi-directional nature of power and its contradictory effects creates an ‘ambivalence of power’ (Butler 1997).

There is more than power relations involved in resource access, use, control, ownership, and conflict. There are also embodied emotions, within what has been called *emotional geographies*, which constitute how nature–society relations are lived and experienced on a daily basis (Sultana 2011). Power and rights of use evolve and can be subverted or enhanced when rules and exchanges are negotiated between actors and users. This dynamic co-evolution is framed by a complex bundle of power structures, under what Ribot and Peluso (2003) call *webs of power*, a concept which describes a wide range of social relationships that constrain or enable actors to derive benefits from resources and involves the empirical ‘mapping’ of institutions, processes and relationships (Ribot and Peluso 2003; Sultana 2011).

As constituents of webs of power, various social determinants (gender, age, class, race) intersect in often unpredictable ways, shifting fields of power at different levels and scales (national, local, intra-household) (Nightingale 2019). Hence, norms and values governing access to trees are not only driven by economic reasons but also perform social, political, and ritual purposes, representing kinship, power relations and spiritual harmony (Peluso 1996). Trees, conceptualised as objects, sites and symbols are at the centre of material and ideological struggles. They can be instruments of power, but also tools of empowerment for different actors (Rocheleau and Ross 1995). This role of trees, or other non-human landscape elements, are intangible, but in fact play crucial roles in the webs of power, shaping the trajectory of social and ecological change.

Against this complex background, the concept of Cultural Keystone Species may help provide a starting point to deepen our understanding of the dynamic social and cultural roles of trees (Garibaldi and Turner 2004). Cultural Keystone Species are known to be essential to maintaining cohesive social relationships within a social–ecological system and without them the society they support would be quite different (Coe and Gaoue 2020; Cristancho and Vining 2004; Nuñez and Simberloff 2005; Platten and Henfrey 2009). Analysing social–ecological interactions around an important tree species such as néré, can give insights into its complex and multiple roles in the landscape and its potential to be classified as a Cultural Keystone Species. This includes the role it plays in identities, cultures, and traditions and in building and enhancing environmental knowledge, narratives, rules and values (Garibaldi and Turner 2004).

Whilst the role of human agency in relational networks is well studied (e.g. as broker, enroller, weaver, or connector), the agency of non-humans, either as material objects or in their cultural socially constructed meaning, is a relatively new field of research in sustainability studies (van der Leeuw 2008). There have been calls for researchers to increase consideration of non-human agency in social–ecological systems (Jones and Cloke 2008), calling for a paradigm shift to deepen analysis of ways humans frame and live out their relations with different species (Pascual et al. 2021). One perspective is the Actor-Network Theory (Latour 1993), which considers how different agents interact cognitively and materially. This theory has now integrated non-human agency and rejects the perspective of humans as distinct from nature (van der Leeuw 2008). It is crucial here to recall that this seemingly recent shift amongst researchers has, in fact, long been entirely integrated in traditional ecological knowledge and embedded within indigenous ontologies and in the work of many indigenous researchers and thinkers (Watts 2013; Todd 2016; Yunkaporta 2019).

In this paper, we explore the social–ecological interactions around the néré tree in West Africa. Several theories and hypotheses have been proposed, for example in ethnobotany, to understand the patterns and processes of human interactions with culturally important species (Gaoue et al. 2017, 2021). Here, we contend that local trees play a key role in structuring interactions within human societies and that intersectional determinants (age, gender, ethnicity) and power distribution shape the interactions with and around those trees.

Our objectives were to: (1) understand the patterns and dynamics of people-ecosystem interactions through the use and management of the néré tree; (2) develop an approach to understand how rights and benefits are shared by people and how power asymmetries are revealed by those interactions; and (3) understand how the néré tree catalyses social interactions and how these are linked to power relationships and (4) reflect on the concept of Cultural Keystone Species in the West African context. We use empirical data on the local history, livelihoods, tenure and rights, social-cultural practices, and natural resource management to document the social–ecological system related to néré. We map and explore this system following a social–ecological network analysis approach (Bodin et al. 2019) and we analyse how rights and benefits are transferred through the system across gender, ethnicity and age (Djoudi et al. 2016). We discuss how the néré tree shapes particular cultural landscapes, where identities and imaginaries are projected in everyday life.

With our analysis of how trees and their agency co-constitute places, culture, and relationships, we aim to contribute to a change of paradigms in global social-environmental agendas, for example landscape restoration and reforestation agendas, in which African cultural landscapes are oversimplified. We also aim to advance discussions on theoretical frameworks of people’s interactions with trees, particularly the roles and use patterns of culturally, historically and environmentally valuable trees, including Cultural Keystone Species.

### Néré in the West African agroforestry systems

The parklands of Burkina Faso are characterized by scattered mature trees, such as shea and néré trees on cultivated fields. Trees have been retained when the woodlands were cleared for crops because of their multiple contributions to people (e.g. food, medicines, cultural value, fodder, gums, fibre, spices, fuel and construction timber). The trees also provide ecosystem functions and services important for human wellbeing and adaptation to climate change, including hydraulic lift, soil fertility, and water permeability. These ‘adaptation services’ (Lavorel et al. 2015) provided by trees are crucial to people’s resilience in a landscape known for its high climate variability (Garrity et al. 2010; Koffi et al. 2017).

Néré, or African locust bean (also known as arbre à farine, fern leaf, irú, monkey cutlass tree, two ball nitta-tree, and nété) is a leguminous perennial deciduous tree belonging to the family Fabaceae. Néré occurs in a geographic range between 5° N and 15° N, from the Atlantic coast in Senegal to southern Sudan and northern Uganda (Hall et al. 1997). The latitudinal belt is widest in West Africa (maximum 800 km) and narrows to the east (Sina and Traoré 2002).

The néré pods contain pulp and seeds (beans), both used as foods. The seeds are crushed, fermented and moulded into balls to make *soumbala*, a fermented and tasty strong-smelling condiment which can be kept for long periods in traditional earthenware pots. It is used in traditional soups and stews, eaten with sorghum- or millet dumplings or porridge. In addition, the farinaceous sweet pulp surrounding the seeds is eaten fresh or made into sweetmeats and drinks. (Campbell‐Platt 1980). Traditionally, locust beans are harvested by women and *soumbala* is prepared for domestic use. These activities have increased in economic and cultural importance, as has the presence of groups of women entrepreneurs involved in the production, trade and distribution of *soumbala*. Although first documented by Michael Adamson in 1757, the use of fermented locust beans dates back at least to the fourteenth century (Sina and Traoré 2002). *Soumbala* is widely traded in the region and increasingly exported to the West African diaspora overseas. It is important in West African culture as it plays a role in all major rituals, including those associated with birth, baptism, circumcision, marriage and death.

*Soumbala* is a valuable source of protein, essential amino acids (e.g. lysine), vitamins (e.g. riboflavin and vitamin C), essential fatty acids and minerals in a region in which nutritional status is a critical determinant of human health (Campbell‐Platt 1980). There is high nutritional value in traditional fermented protein foods, particularly in regions where people depend on carbohydrate staples such as yams and cassava. There is substantial evidence that fermented foods have major benefits for human health, particularly via their influence on the microbiome of the human gastrointestinal system and in stimulating the development and maintenance of a healthy, effective immune system (Klaenhammer et al. 2012; Maslowski and Mackay 2011).

The tree also supplies medicines and fodder, and its leaves are incorporated into soil as green manure. It is also important in apiculture, being a good source of nectar and suitable for the placement of beehives. Néré trees, as nitrogen-fixing legumes, enhance soil fertility and the yield of nearby crops. The trees also provide shade and shelter for people and livestock (Sina and Traoré 2002).

## Materials and methods

We collected data using mixed methods to explore the social–ecological interactions around néré with a network analysis. The detailed steps of the network analysis, along with data and R functions, are described in Supplementary Information (SI).

### Fieldwork sites

**Fig. 1:**
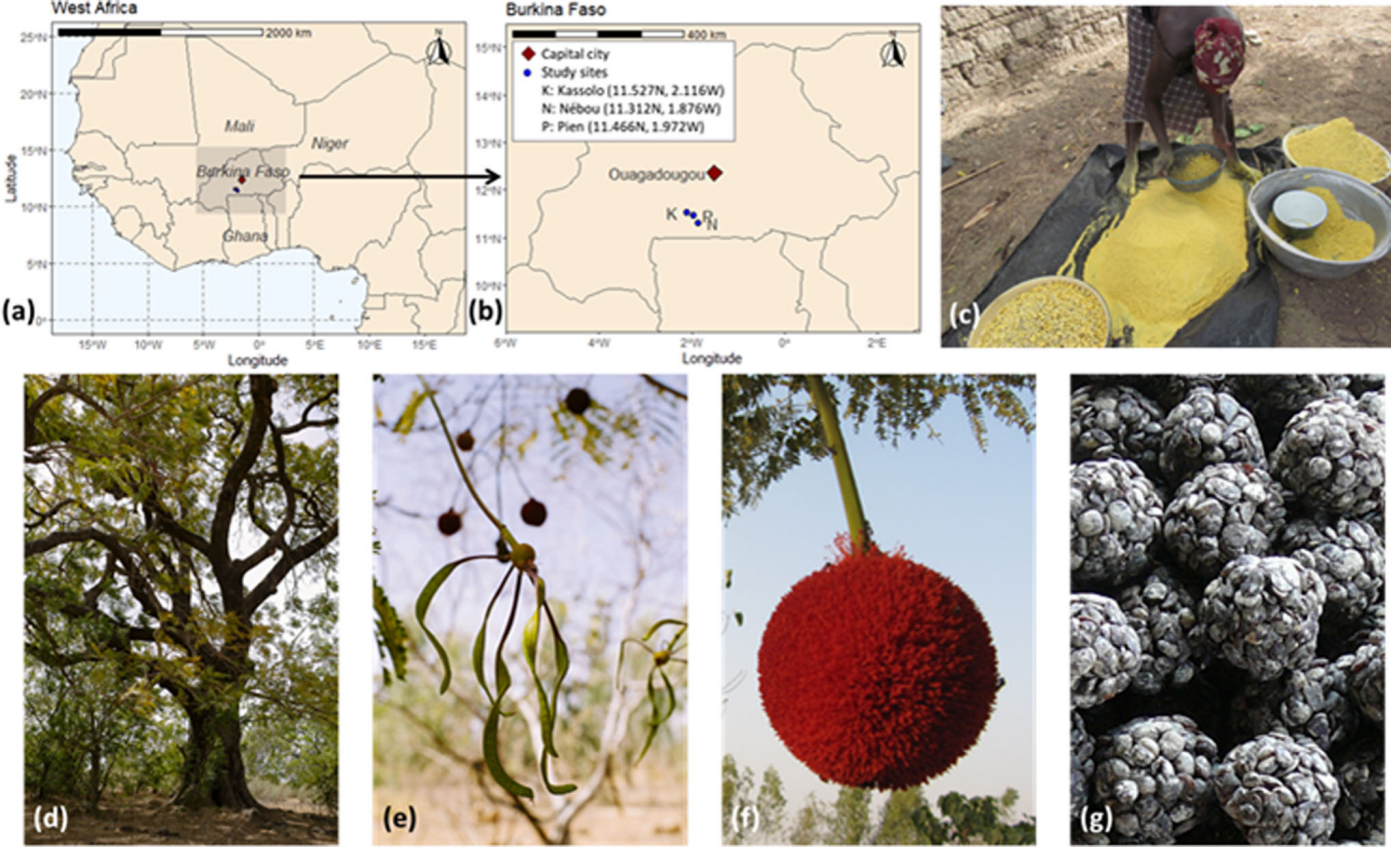
Location of the study sites in West Africa (**a**) and Burkina Faso (**b**). Pictures of Néré: processing of its pulp (**c**), tree (**d**), pods (**e**), flowers (**f**), and soumbala (**g**) (all pictures by CIFOR)

The three villages included in the study are located in the Ziro and Sissili provinces in central-west Burkina Faso (Fig. 1). The study sites are within the South-Sudanian ecoregion with rainfall between 800 and 1100 mm (from June to September). The vegetation is characterized by a dry forest and shrub and tree savannah. Néré is found in cultivated fields and fallow lands, together with other important agroforestry species such as the shea tree, *Lannea microcarpa* Engl. & K. Krause (wild grape) and *Tamarindus indica* L. (tamarind). Néré products play an important role in nutrition and livelihoods at the study sites; it is frequently consumed by most households across ethnicities (Teklehaimanot 2004; op et al. 2012; Vinceti et al. 2018; Pehou et al. 2020).

The main ethnic groups in the region are Nouni, Mossé and Fulani. The Nouni are considered the original inhabitants. They are custodians of the land and are responsible for related customs and practices. Chiefs of land are responsible for religious matters (e.g. rituals and sacrifices) and land issues, whereas village chiefs have supreme authority on land decisions, as well as political issues (Lentz and Sturm 2001).

The Mossé and Fulani migrated into the area over the past four decades. The Nouni and Mossé are farmers who harvest néré products, whereas the Fulani are traditionally nomadic and semi-nomadic herders who have become sedentary and now cultivate the land and harvest néré products in addition to their livestock raising activities (Howorth 2019). The Nouni people, considered autochthonous to the area, represent less than half of the population, whilst Mossé and Fulani migrants represent 50–90% of the population, depending on the village, with a predominance of Mossé (DGAT 2010) (more details in Pehou et al. 2020). Herein, for the ease of the reader in differentiating these ethnic groups, we use generic names, acknowledging they do not fully reflect original identity. Nouni are called ‘native farmers’; Mossé, ‘migrant farmers’ and Fulani, ‘pastoralists’.

Land is controlled by men and trees are owned by the head of the household in the lineage that first settled the area. Although trees are under the control of men, use rights to néré products are held by women, who organise the harvest of néré pods and activities related to processing and sale. There is a distinction between native farmer householders, who have secure access rights to land, and migrant farmers and pastoralists. In the customary tenure of the native farmer group, land is under control of the family lineage: each male descendent is entitled to a portion of the land. In contrast, a male head of a migrant household can borrow land from a native under traditional arrangements, but borrowers cannot use trees that were already growing on the land, only those established after they borrowed the land. Migrant farmers are increasingly purchasing land and obtaining full rights. Woodlands that are considered common property are derived from very old fallows and pastures (Pehou et al. 2020). The patrilineal customary regimes are similar for all ethnical groups considered in this study. Women do not own land, cannot inherit land, and cultivate much smaller plots than their male counterparts (1 ha versus 3 ha on average, respectively) (Ouédraogo et al. 2005).

The data used in this study came from various research projects conducted between 2013 and 2017 on land and tree tenure, value chains, and livelihoods related to néré, in which two authors of this paper participated (Pehou et al. 2020). The study involved national research partners and scientists originally from the Nouni community. Before data collection, community members, traditional leaders and all participants were informed about the objectives of the research. They were explained that their participation was voluntarily and that all information would be treated confidentially. The researchers obtained the oral consent of all participants before the start of interviews, surveys or group discussions.

Our qualitative analysis is built on a synthesis of a rich dataset collected by four means. First, ethnographic and participant observation and 36 qualitative interviews (six women and six men selected from each of the three socio-ethnic groups: Mossi, Nouni and Fulani) were used to gather data on the cultural, social and spiritual context, land tenure, and tree access rights. An interview guide with open-ended questions was used to understand the individual and collective management of the néré trees and products, and its difference according to social groups (differentiated by gender, ethnicity and type of livelihood). We documented land tenure and tree tenure regulations and restrictions. Through 18 focus group discussions, we collected information on the actors involved in néré activities, their relationships and exchanges, the importance of néré for different social groups, the different types of fields and woodlands where it was harvested, the products used, the seasonality of use, and the threats to the species. Three groups of women (10) and men (10) were formed with migrant-Fulani herders, indigenous-Nouni farmers, and migrant-Mossé farmers. The data were documented via field notes. In addition, the third author of this study, originated from the region, resided in the selected villages during the néré harvesting season in April and May 2013. Direct observation and informal discussions resulted in additional information about the organization of the harvest activities in the fields, fallows, and woodlands. This approach is generally considered effective to understand the daily routines and the subtleties in the rules and knowledge about the tree (Albuquerque et al. 2014). The participant direct observation during the harvesting season also made it possible to triangulate the information gathered through other means (e.g., focus group discussions) and to clarify some complexities in néré rights, access, and uses.

Secondly, we interviewed 180 women, randomly selected across ethnic groups: 62 Nouni, 81 Mossé, and 37 Fulani (these numbers are proportional to the population of each ethnic group across the selected villages). The semi-structured interviews dealt with the use of the néré tree, the economic value of different néré products, the economic and social exchanges around néré, and the participation of household members in its management, harvest, and transformation activities.

Thirdly, we analysed the history of the sites to understand the evolution of access rights and changes in harvesting and use practices. For this, we listened to the life histories of six women aged 55 years or older and conducted semi-structured interviews with customary authorities, official authorities and technical staff from the Forest Service.

Finally, we used data from a survey of 280 néré traders and 133 consumers in 24 selling sites including traditional markets, food stores and shops around the study area and in the capital city of Ouagadougou to understand the néré product markets. The survey targeted all actors, both men and women, who sold at least one of the four néré products (pods/fruit, seed, flour/pulp, and soumbala) in those selling sites.

### Network analysis

We conducted a social–ecological network analysis, a method based on graph theory, to understand the structure of the interactions around néré. Network graphs are mathematical structures used to model pairwise relations (links) between objects (nodes) (Janssen et al. 2006). Social network analyses have been used to explain interactions between actors or institutions. For example, they have been used to determine which actors hold power or how interactions (e.g. coordination, competition, information sharing) may influence the governance of natural resources (Bodin and Crona 2009; Locatelli et al. 2020; Vallet et al. 2020). Recent developments have explored patterns of relations in social–ecological networks by adding an ecological dimension to social network analyses (Janssen et al. 2006; ; Bodin and Tengö 2012; Bodin et al. 2019; Sayles et al. 2019).

**Fig. 2:**
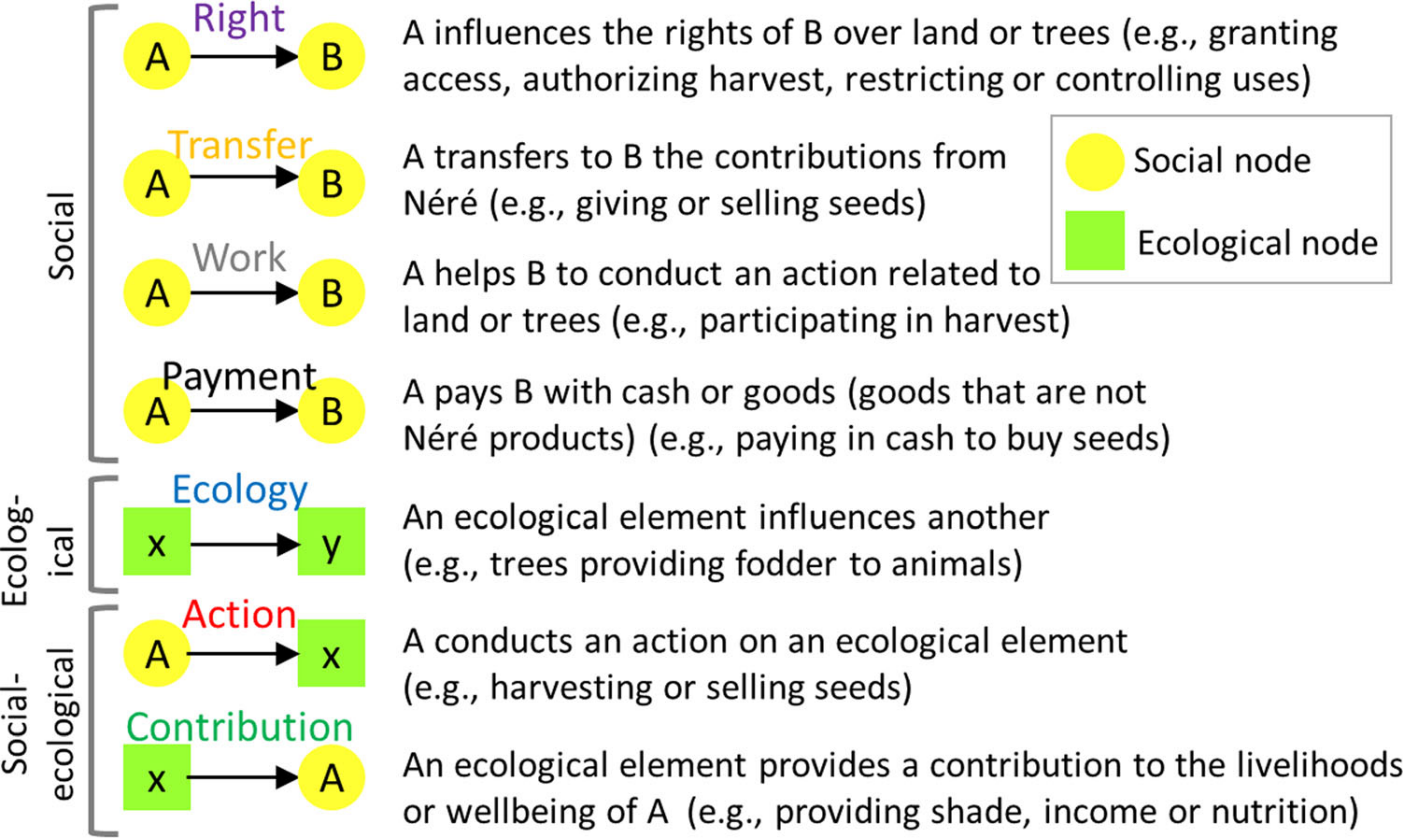
The seven types of links (social, ecological, and social–ecological) in the néré socio-ecological system

The qualitative field data were summarized visually by drawing an initial network of social–ecological interactions, including how rights and benefits are captured and transferred. We defined the key ecological nodes (land, animals, trees, seeds on trees, and harvested seeds) and identified the social nodes (actors related to néré). The list of social nodes included local inhabitants (described by their gender, age, and ethnicity) and external factors (described by their roles, such as traders or consumers). At the study sites, as households can be monogamous or polygamous, our analysis distinguished between first spouses and subsequent spouses because each has a different role and rights. We drew the links between the nodes using the software Visual Understanding Environment (Tufts University 2015). We examined the density of the network (the ratio of the number of links to the total number of possible links) to compare the number of interactions at the level of the land, tree, and product. We then coded the links into seven types (Fig. 2) and created a second network with the same information as the first, but mathematically formalized as a multilayer directed network. ‘Directed’ means that links between nodes have a direction and ‘multilayer’ means that we consider multiple types of relations as layers of the overall network (Kivelä et al. 2014; Dickison et al. 2016).

The resulting network was described first by identifying nodes with highest degrees (i.e. number of links from or to a node) and inventorying the existing combinations of links between pairs of nodes. Secondly, we plotted the network using a force-directed layout algorithm (Fruchterman and Reingold 1991), in which two nodes are close to each other if they are linked (which is visually depicted as a group of connected nodes that are in the same region of the plot).

We then focused on two sub-networks, both built using the full set of nodes and a subset of selected links. The first one, called ‘network of rights’, showed how people exchanged rights amongst themselves and acted on ecological elements (selected links were “Right” and “Action”). The second one, called ‘network of benefits’, showed how ecological elements interacted and produced contributions to people (e.g. supplying seeds) and how people interacted to exchange these contributions (e.g. giving seeds) (selected links were “Ecology”, “Contribution”, and “Transfer”). We drew these two sub-networks and analysed their hierarchical nature. A hierarchical network is one in which paths (sequences of links) are not reciprocated, for example, if node A influences node B, node B cannot influence node A, directly or indirectly through other nodes. Hierarchical networks show the pyramidal structure of a group, in which dominant actors can be identified.

We calculated a simple index to identify the degree of hierarchy of the full network and the two sub-networks (the Krackhardt hierarchy score, which has a value of 1 for a fully hierarchical network and 0 for a non-hierarchical one) (Krackhardt 1994). In the hierarchical sub-networks, we identified the dominant actors, i.e. the nodes that could reach a large number of other nodes following the directed links of the network. In the sub-network of rights (respectively benefits), the dominant actors would be the nodes that can transfer rights (benefits) to many other nodes, directly or indirectly. The two sub-networks were plotted using a layout algorithm for hierarchical graph drawing (Sugiyama et al. 1981), in which the most dominant actors are at closest to the top. Networks were created and analysed in R version 3.6.2 (R Core Team 2019) with the *igraph* package (Csardi 2018). Data and R script are freely available (Locatelli 2022).

## Results

The result section focuses on the findings of the network analysis and illustrates them with narrative descriptions of qualitative data collected in the field (called “stories from the field” hereafter).

### Full network

**Fig. 3:**
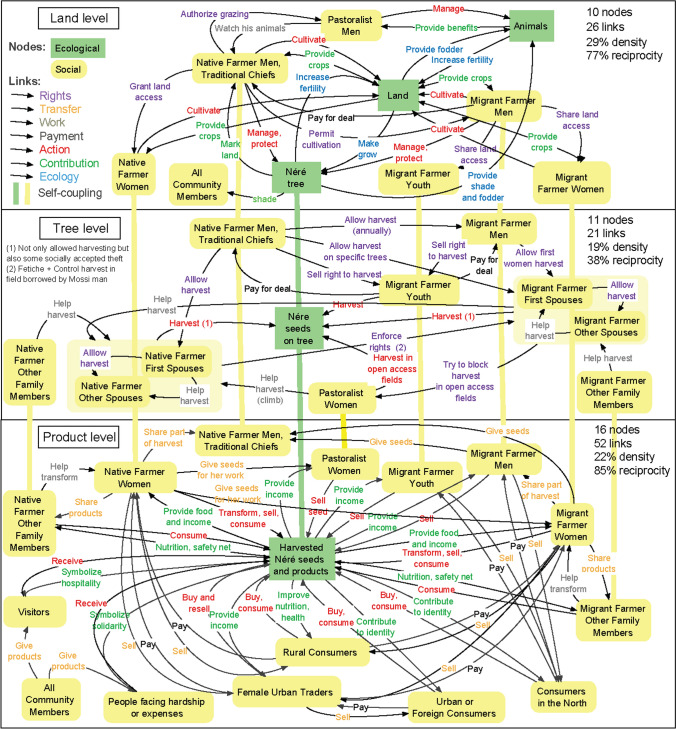
First drawing of the full network, with links around néré taking place at the level of the land (e.g. links amongst animals, land, and trees, or links amongst land managers and trees), the tree with its seeds (e.g., people harvesting seeds), or the harvested seeds (e.g. people transforming seeds or selling products). The vertical self-coupling links are between the same node at different levels. Four indicators are given at the top-right corner of each level: number of nodes, number of links, density (number of links divided by the total possible number of directed links), and reciprocity (percentage of the number of links having a reciprocated link)

The first drawing of the network (Fig. 3) revealed a high diversity of actors interacting with and through néré trees by managing trees, harvesting trees, exchanging products (pods, seeds, and *soumbala*) and benefiting from different products or services. The three levels of the network (land, tree, and products) showed differences in terms of gender and ethnicity of the actors involved. There were more male than female social nodes at the land level, where male nodes represented 67% of all nodes with an identified gender (e.g. migrant farmer man or woman). The opposite was observed at the tree level (62% female nodes) and the product level (57% female nodes), which confirmed the adage that often came up amongst the interviewed men: "*the néré is the woman's work*”. Pastoralist men interacted with other nodes only at the land level, whereas pastoralist women interacted with other nodes at the tree and product levels.

This network confirmed the multiple stories from the field about how néré produces multiple benefits including food and nutrition, soil fertility conservation, safety nets in times of hardship, and non-material benefits such as symbolic meaning and identity building, connection to place for distant consumers, and an expression of solidarity and hospitality towards visitors and people in hardship. The network also showed the dense web of links around néré amongst social groups, like farmers and herders of different ethnicities, men and women of different ages, migrants or native people with different histories and duration of settlement in the site. An example story from the field is about pastoralist women, who are not granted access to néré and offer their harvesting skills and services to mainly elder native farmer women with rights to harvest néré, in exchange for néré seeds. This interaction has resulted from a renegotiation and evolution of rights as pastoralist women were originally excluded from accessing néré. Beyond this mutually beneficial relationship during the harvest season, this practice strengthens social capital between two groups, which are in very different social positions.

The network showed differences by levels (Fig. 3). There were more nodes at the product level (*n* = 16) than at the tree (11) or land (10) levels. The high density of links in the network (between 19 and 29% depending on the level) revealed the multiplicity of social and social–ecological interactions around néré. The higher number of links at the product level (*n* = 52) showed a fine-tuned system of sharing and reciprocity amongst diverse actors.

**Fig. 4:**
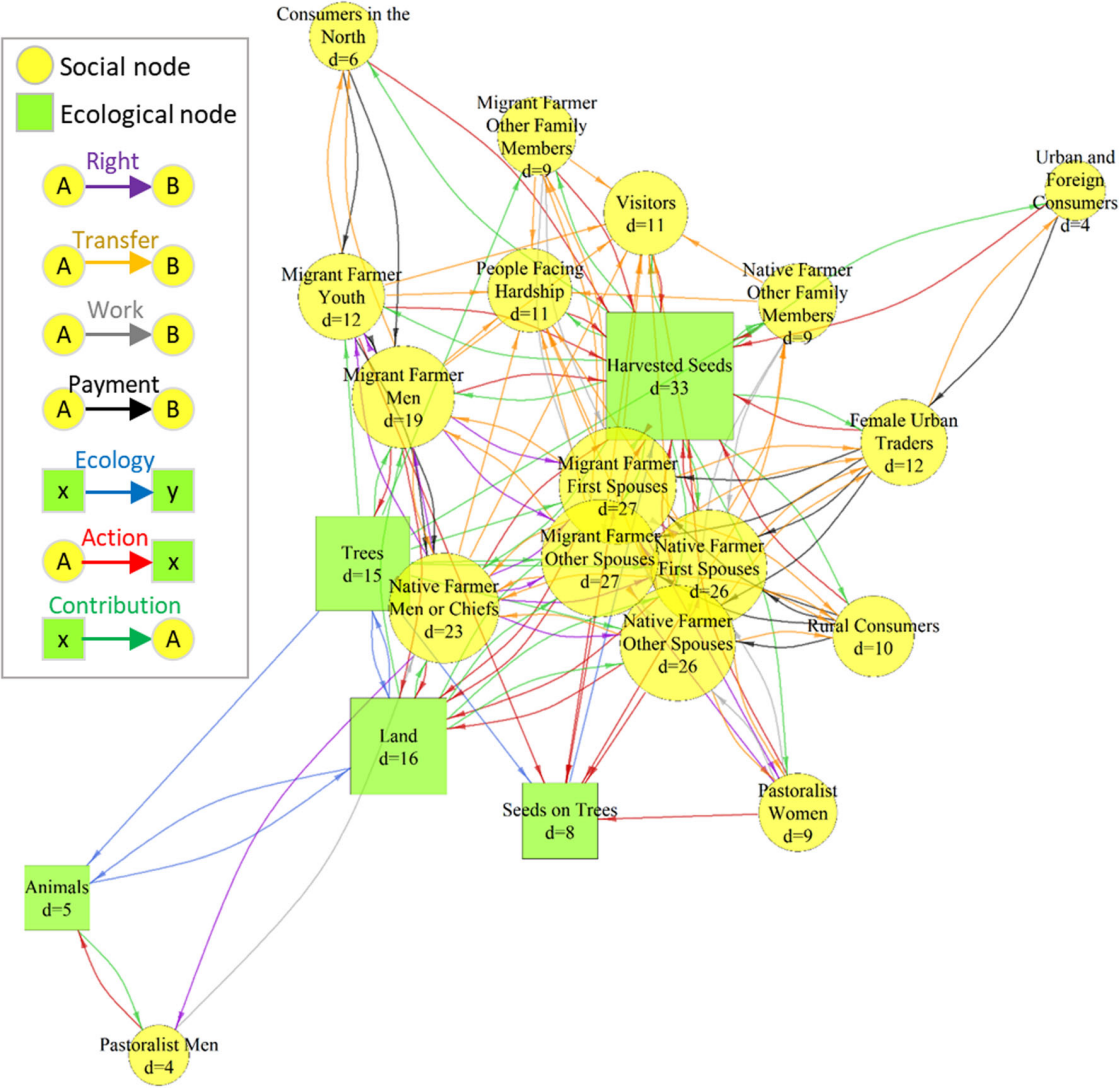
Mathematically formalized representation of the multilayer directed network around néré (the area of each node is proportional to its degree and the node label shows the node degree d)

In the network mathematically built from the drawn network (Fig. 4), the “harvested seeds” node had the highest degree, which was expected given the density of interactions around néré products. The social nodes with the next highest degrees were four female nodes (first spouses and other spouses of migrant farmers and native farmers) and one male node (male native farmer or traditional chief).

## Links between pairs of nodes

**Fig. 5:**
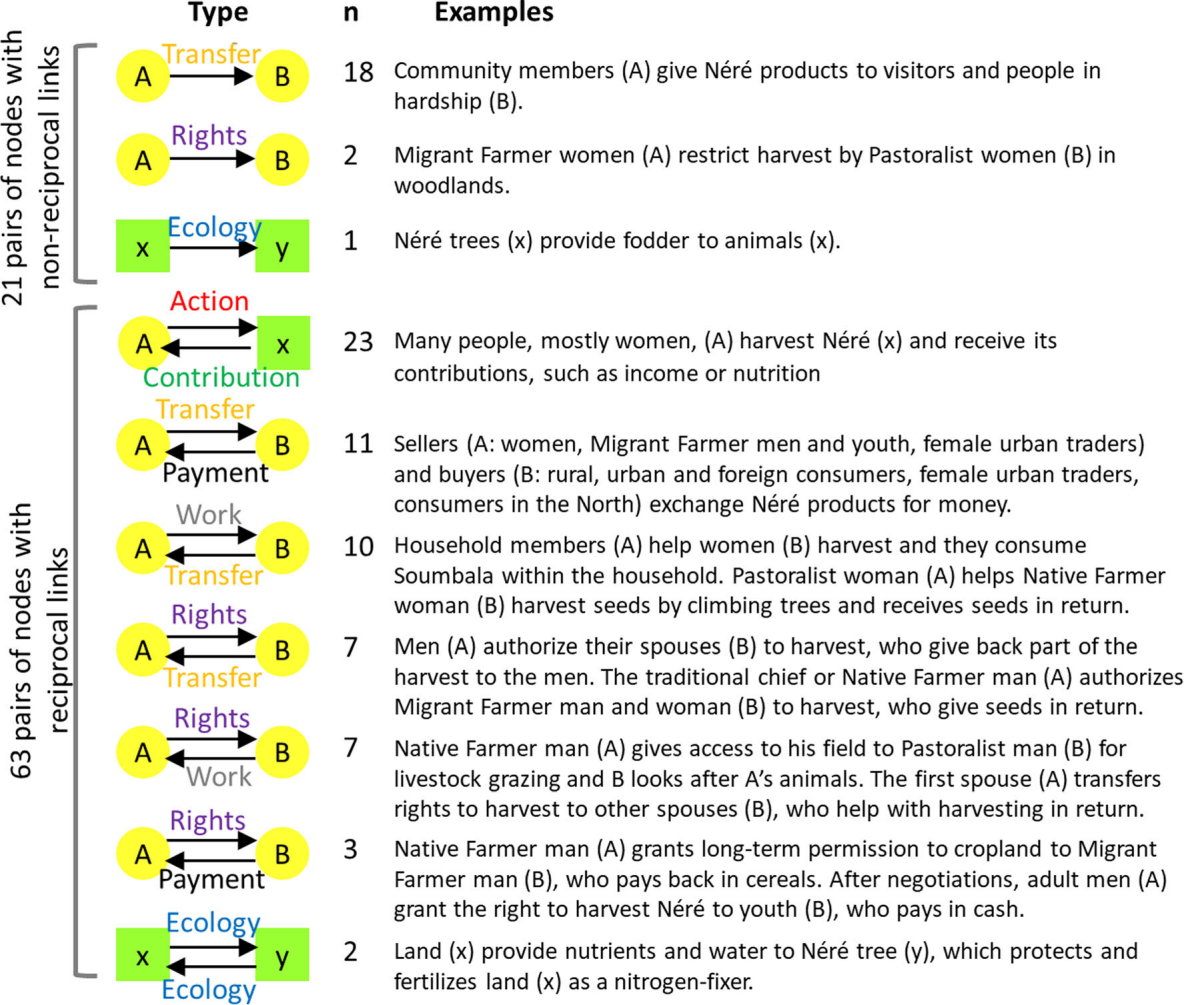
All types of observed interactions between pairs of nodes and their number of occurrences in the network (*n*)

The full network was completely non-hierarchical (Krackhardt hierarchy score = 0), because of the multiplicity of reciprocal links. Amongst the 84 pairs of connected nodes, 63 had reciprocal links (Fig. 5). Non-reciprocal links between ecological nodes generally formed triangles (e.g., land provides nutrients to trees; trees provide fodder to animals; animals fertilize land), which created indirect reciprocity and positive feedbacks for the production of benefits from néré. Other ecological feedbacks involve agriculture, as cultivation beneath the néré trees creates synergies that are typical of agroforestry (e.g. regulation of fertility, shade, microclimate, and soil moisture).

The reciprocity-based associations between farmers and pastoralists, typical of Sudano-Sahelian social–ecological systems, underpin many ecological relationships in the network. An example story from the field is about farmers providing access to land and fodder to pastoralists, whilst pastoralists improve farming productivity through provisioning of animal manure. These multiple win–win interactions contribute to the resilience of social–ecological systems but have undergone significant changes in recent decades. Because of the development of commercial and more intensive agriculture, fertilization with animal manure and urine has been progressively substituted by industrial fertilizers, which are subsidised in the case of cotton production. As a result, the need for animal manure has declined and “*access to pasture in agricultural fields for grazing animals has not only become scarce, but animals are probably exposed to contamination by these chemicals*”, as argued by a pastoralist. The longstanding relationships between farmers and pastoralists have shifted from co-operation to competition for land.

Amongst the non-reciprocal links between social nodes (*n* = 21), 18 were unidirectional transfers of néré products from community members to visitors and people in hardship, as described by a fifty-year-old Nouni woman from the village of Nébou: “*the soumbala I bring to my daughter, who is newly married, enables her to save money to meet her household’s other needs*”. In addition, stories from the field suggested that there is reciprocity at a higher outlook, as those who give seeds today may one day be on the receiving end of such a link. The other non-reciprocal links between social nodes (*n* = 2) were about rights, with women restricting néré harvest by other women, through direct control or the deployment of fetishes (objects believed to have supernatural powers) to ward off unauthorized harvesters. Yet, although our network did not show reciprocity here, there was some form of counter power that could make this a bi-directional interaction (e.g. resistance to restrictions, negotiated deals, and use of ‘counter-fetish’ objects).

Many reciprocal links between pairs of social nodes were market exchanges (i.e., transfer and payment in Fig. 5) or work paid with néré products (i.e., work and contribution). All the others were about sharing rights to néré (rights to harvest or rights over products) in return for work, a payment, or néré contributions (Fig. 5). Some reciprocal links also referred to relationships at a higher level, for example, exchanges between migrant and native farmers as an expression of long-term relationships and solidarity between ethnic groups.

The stories from the field showed that some interactions have been evolving, for example, male migrant youths have increasingly acquired harvesting rights through monetary transactions. The migrant youths purchase rights to néré pods from native farmer men whilst pods are still on the trees. The migrant youths transport and sell them in their villages of origin in the dry north of Burkina Faso, where néré trees do not grow. In this way, they build new social networks or revive old social ties, including with family. However, this change in harvesting rights reduces seeds available for harvest by some women. Hence, over time, changes in one social node may affect interactions of other social nodes and gains or losses of rights and benefits cascade through the entire network.

### Cascade of rights and benefits

**Fig. 6:**
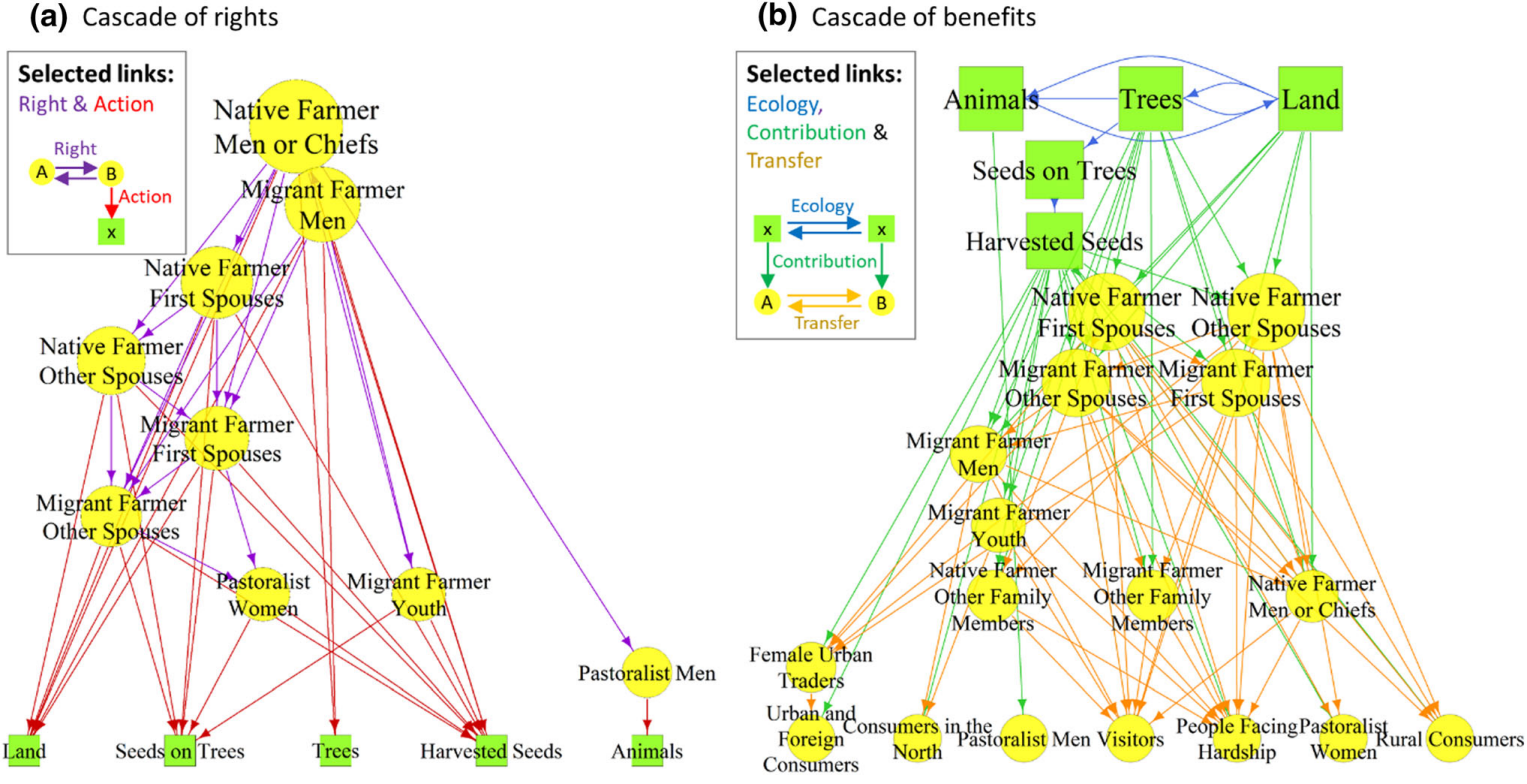
The two sub-networks representing a cascade of rights (**a**) and a cascade of benefits (**b**). The link selected for each sub-network are shown in the top-left box. The dominant nodes are located towards the top. Node size is proportional to its dominance

The first sub-network (‘network of rights’), which included the exchanges of rights amongst people and the actions of people on ecological nodes, was fully hierarchical (Krackhardt hierarchy score of 1) (Fig. 6). Hence, we refer to it as a ‘cascade of rights’, with ‘cascade’ demonstrating a unidirectional flow from the top to the bottom. A very high degree of hierarchy was also found within the second sub-network (Krackhardt hierarchy score of 0.98), which included ecological links, the contributions of néré to people, and transfers of these contributions amongst people. We refer to this ‘network of benefits’ as a ‘cascade of benefits’.

The nodes at the top differ strikingly between the two sub-networks. Whereas rights generally flowed from men to women, native to migrant farmers and pastoralists, and the old to the young, the social nodes at the top of the cascade of benefits were women (first native, secondly migrant) and benefits flowed from women to men. The ecological nodes had different positions in the two sub-networks. This corresponds with our initial conceptualization of the two sub-networks and their links, wherein the ecological nodes were necessarily at the bottom of the cascade of rights and at the top of the cascade of benefits.

In the cascade of rights, native farmers were at the top. Indeed, the male heads of households from the lineage that first settled in the area (native farmer man) retain all rights with respect to néré trees, including the right to selectively protect, plant or cut individual trees. Husbands transfer the rights to harvest to their spouses. Women's rights to harvest néré depend on their migration status, ethnicity, and position as first or subsequent spouse. The first spouse in a native or migrant household receives rights to harvest specific trees from the native farmer and can then share part of this right with the other spouses. Other women in a lineage can get access by helping during different tasks, from harvest to the processing of the seeds. Harvesting can also occur without clear rights. A frequently told example story from the field is about ‘tolerated theft’, which is an informal and subtle social norm that transcends rules: it is widely accepted that some women will harvest some pods without acknowledged permission or traditional rights.

In the cascade of benefits, women were at the top. Although trees are under the control of men, women are the ones who organize and manage the harvest, manufacture and sale of néré products. Most flows of néré products and their benefits are instigated by women, whether within a household (e.g. a woman sharing part of the harvest with her husband or other co-wives), within the community (e.g. a native woman giving seeds to a pastoralist woman for her help in harvesting), or outside (e.g. female traders selling soumbala in the city).

**Fig. 7:**
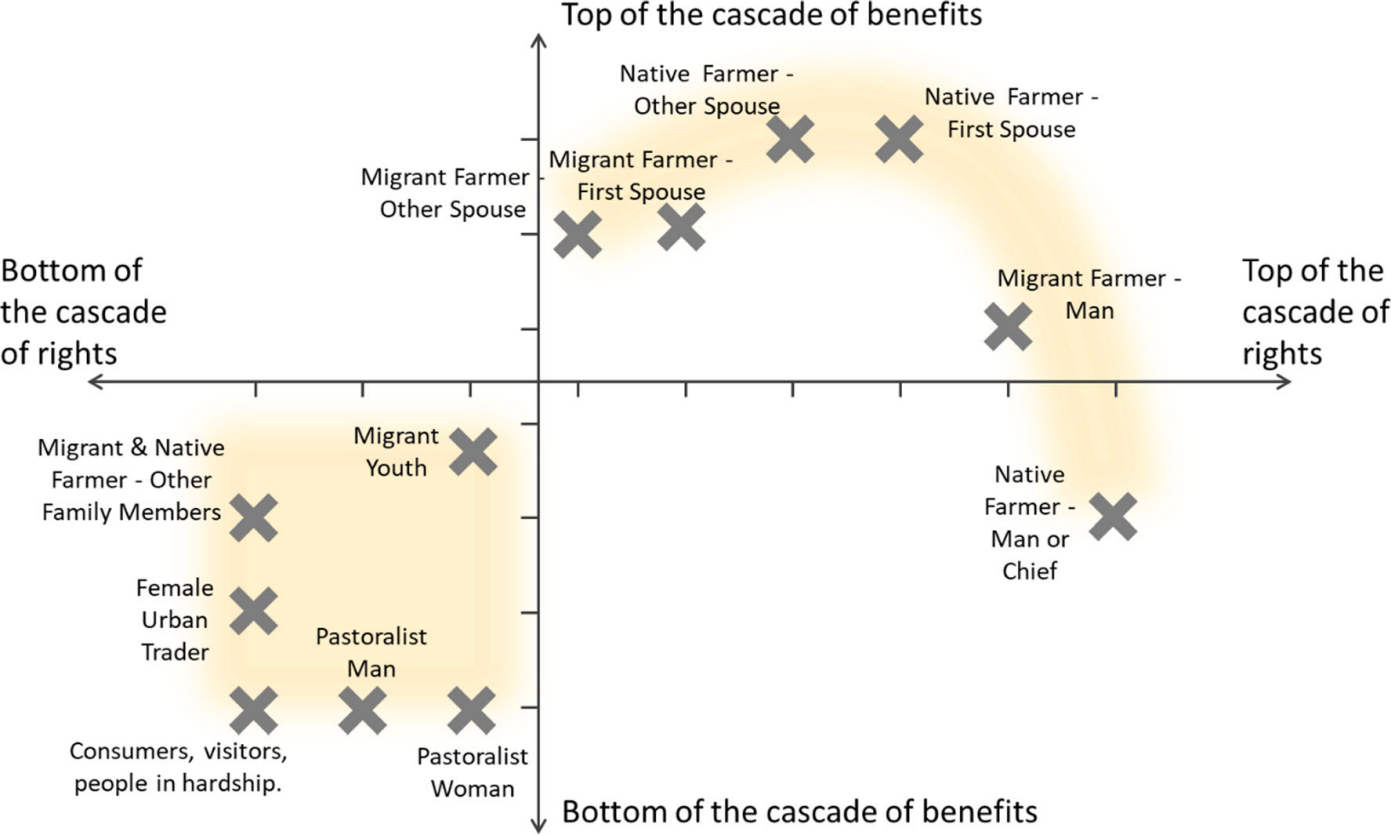
Positions of social nodes in the two cascades

The position of the same actors was different in the two cascades (Fig. 7). Some actors were at the bottom of both cascades: pastoralists, youths, traders, consumers, visitors, and people in hardship. Other actors with top positions in one of the two cascades (i.e., native and migrant farmer men and women) were situated towards the middle of the other cascade. For example, native and migrant farmer women were high in the cascade of benefits and towards the middle of the cascade of rights. Conversely, native farmer men were at the top of the cascade of rights, but towards the bottom of the cascade of benefits.

## Discussion

Our network analysis helped understand the social, ecological, and social–ecological interactions around néré, a native tree in West Africa. We demonstrated that social–ecological network analysis is a useful approach for understanding social and ecological interactions surrounding culturally important species such as néré, whilst describing how rights and benefits flow. The network brings a broad picture of all relationships and rights, beyond a simple perspective of the ‘haves’ and ‘have-nots’. Because we recognised that our network analysis presented a stylized and static view of the social–ecological system and overlooked its dynamics, we complemented the network analysis with narrative descriptions based on examples of interactions and changes in relationships over time.

A limit of our method lies in a certain degree of subjectivity in the translation of qualitative data into network nodes and links. One option to improve the method for future studies could be to systematize network development through the use of text coding tools or a systematic translation of qualitative information, for example with Unified Modelling Language (Müller and Bommel 2007). Another option would be to analyse which nodes and links were the most often cited by key informants (e.g. inhabitants or external experts). This approach could help detect the core rules of néré management and the secondary ones (e.g. the socially tolerated 'theft'). An improvement to our study could be to build networks in which social nodes are individuals rather than types of individuals. The resulting larger set of nodes would allow hypothesis testing (Gaoue et al. 2021). The rights of individuals, their harvest or use practices, and their knowledge about néré could be analysed to test hypotheses on the flows of rights, benefits, and information (Reyes-García et al. 2013; Gaoue et al. 2017).

Our study combined formal mathematical methods with reflexive ethnographic approaches and qualitative empirical data. Our interdisciplinary work tried to traverse boundaries in socio-ecological studies and overcome binary divisions, for example between qualitative and quantitative approaches and between social and ecological or human and non-human elements of the landscape. We argue that, rather than representing irreconcilable methodologies, when juxtaposed thoughtfully, network analysis and stories from the field can shed new light on socio-ecological systems, despite being rooted in distinct epistemologies.

### A tree that builds social relationships across diverse groups

The density of the network shows that the néré is not only a provider of ecosystem services but acts as a broker of social interactions, creating and shaping social relations of exchange, reciprocity, inclusion and exclusion through which diverse identities, social positions, and landscape histories interact. Two factors stand out from our analysis, illustrating the role of néré as a broker of social interactions. First, there is the intersectional diversity of actors across genders, ethnicities, and age, all connected through the tree. Indeed, the cascades of rights and benefits clearly show that interactions through néré are not determined by one single identity but by a combination of different social attributes of settlement history, ethnicity, gender, social class, and marital status (Cho et al. 2013; Djoudi et al. 2016). Thus, power emerges from the interplay of the attributes of each actor and cannot be understood from the perspective of a single category. Secondly, there is the versatility and negotiability of certain rights, since the ability to benefit from néré products goes far beyond an official, fixed access codex to include broader social mechanisms that permit access. These access mechanisms include exchanging skills with privileged groups or building social relations with people who can mediate access, or socially tolerated ‘theft’. These informal and adaptable social mechanisms allow access for community members who would otherwise be denied in the customary rights system.

These findings call for a better social disaggregation in environmental studies, beyond the simplified generalizations of ‘local community’ or, in best cases, ‘herder/farmer’ or ‘women/men’ dichotomies (Arora-Jonsson 2011; Djoudi et al. 2016). As has been noted by other authors working on land issues in Africa (Lund 2011), tree tenure regimes seem to have the similar diversified, finetuned and multi-actor nature as land tenure regimes in West Africa. There is a need to consider a range of issues spanning access to, and control over, natural resources and the distribution of power amongst an intergenerational, inter-ethnic and inter-gender nexus.

The network analysis shows how néré mediates crucial social-cultural linkages that are characteristic of a cultural keystone Cultural Keystone Species, as shown by the high density of interactions and exchanges in the networks, including diverse socially differentiated actors and identities (women, men, pastoralists, farmers, young people, rural and urban inhabitants), and the multiple levels across which interactions occur. Néré plays essential roles in maintaining cohesive social relationships within the West African savannah social–ecological system. It occupies a unique position in this cultural landscape demonstrated by the intensity, multiplicity and persistence of its use and its symbolic role across gender, ethical and intergenerational relations.

Our results suggest that, given its unique position within the socio-ecological landscape, in which identities and cultures and traditions as well as environmental knowledge, narratives, rules and values are enmeshed and (re)produced (Garibaldi and Turner 2004), néré may be a Cultural Keystone Species in Burkina Faso and, more generally, in West Africa. In light of debates about what exactly is and what is not a keystone Cultural Keystone Species, more research is needed to understand whether néré complies with suggested criteria, such as cultural uniqueness and irreplaceability (including the richness and meaning of terminology and names in local languages), intensity and multiplicity of use, symbolism and individual or collective memories, roles in ceremonies, and persistence of use despite cultural changes.

### A tree that balances powers in society

The finding that actors at the top of the cascade of rights were often at the bottom of the cascade of benefits, and vice versa, is striking. It shows that néré is at the centre of exchanges of multi-dimensional power, struggles and empowerment. The two forms of power in the two cascades of rights and benefits evoke possible powers and counter-powers as actors may be simultaneously enabled and constrained by their relative position within the cascade of rights. Whilst some actors at the top of the cascade of rights are conferred clear powers, there are more complexities to this web of power around néré beyond just rigid positions at the ‘bottom’ or ‘top’ of the cascade.

In the néré-related webs of power, some actors, such as native farmers men or chiefs, control access by controlling *bundles of powers* (Ribot and Peluso 2003). But other actors, such as the spouses of the male household members, form *bundles of owners* (Ribot and Peluso 2003) and act in concert to counterbalance these bundles of powers and ensure access. The néré tree is thus a medium to exert individual, territorial, and institutional power as a means for territorialization and anti-territorialization claims.

Seemingly disadvantaged actors have other forms of power different from the power of people at the top of the cascade of rights. This invisible power can be framed using the Resource Dependence Theory (Reimann and Ketchen 2017): as néré products are often in scarce supply, people who give and sell néré have power because they are on the more favourable side of the dependence asymmetry. Another framing, less focused on economic exchanges, comes from the Social Exchange Theory (Blau 1964; Emerson 1976), which describes how helping others (for example, though gifting of *soumbala*) brings social rewards and associated power. Similarly, Mauss’ Social Theory of Reciprocity and Gift Exchange shows how the donor is stronger both socially and emotionally because of the gift given (Mauss 2002).

But, more importantly, there is also the social balancing act, in which the tree plays the role of *power equaliser* to mediate power imbalances between different actors. Whilst power asymmetries often appear rigid, the most appealing finding of our power analysis is an inherent flexibility in the interaction system, which allows different inhabitants to gain and negotiate access at different times and using different assets, abilities and mechanisms. It is important to highlight that this kind of flexibility does not necessarily counteract marginalization, produced at other levels within society and based on other social determinants. The tree balances, but does not necessarily challenge, extant marginalization.

Whilst ‘win–win’ outcomes are in many cases impossible to achieve, such as nuanced rights and benefits, the system creates a balanced social mechanism between access and restrictions. Central to this system is the maintenance of flexibility to address social trade-offs between different groups and individuals and for coping with uncertainties. This finding shows how self-organizing and self-regulating social-political arrangements have vital importance and meaning, particularly in drylands that are characterized by high uncertainty and climate variability and where flexibility is vital for diverse social groups to respond and adapt to unpredictable and sudden change. Furthermore, this complex multidimensional praxis of power is profoundly different from the unidimensional way power is theoretically framed in some environmental studies. Our results call for the analysis of power which goes beyond top-down framing to include an emancipatory formulation of power as ‘positive yet unrealized’ (Morrison et al. 2019), enabling the mobilisation of resources and which can also manifest *innovative power* and the construction of new resources (Kok et al. 2021).

We need to rethink the simplistic dichotomous way in which power is framed. The intersectional power analysis used in this study goes beyond the frequent framing of communities as homogenous groups and illustrates the different types of powers, which can be visible or invisible or can enable emancipation.

### Non-human agency and indigenous traditional knowledge

We started our research from the perspective of a social–ecological network analysis around néré. The more we advanced in our analysis, the more we recognized that social relationships played out not just around the tree but *through* the tree, often driven by it. We encountered different forms and roles that néré takes within the network: tree, pulp, seeds, *soumbala*, shade, fodder, nitrogen fertilization; but also provider, protector, mediator, unifier, and likely Cultural Keystone Species. Néré is an agent, just as are all the people in the network, and its materiality and agency matter greatly. The materiality of néré is an elusive and difficult concept because the tree exceeds its physical properties. It shifts identities, it creates imaginaries and evokes symbols, and it has histories to which we respond.

Referring to what Jones and Cloke (2008) call the *ecologies of interrelating trajectories*, we argue that néré and other native trees shape, link, and bridge multiple cultural spaces, contributing to the unfolding and refolding of nature–society relations. Seen through the lens of power analysis (Ahlborg and Nightingale 2018), human and non-human interactions are embedded in, and shaped by, tangible human agency and the elusive power within institutional, material, and social pressures. This perspective goes beyond two-way relationships (people and environment, human and non-human) toward a web of relationships between different people and identities and non-human elements in the landscape. It enables a re-framing of human-nature relations as ‘human-as-environment’ interactions. Hence, the néré social network is best conceived as a constant process through which human and non-human agents, diverse identities and imaginaries forever weave themselves into the fabric of their natural, social, and cultural worlds.

It is important to acknowledge Indigenous Peoples for “their millennia of engagement with sentient environments, with cosmologies that enmesh people into complex relationships between themselves and all relations” (Todd 2016, p. 6). Furthermore, Indigenous thinkers argue that agency has erroneously become exclusive to humans, removing non-human agency from what is considered to constitute a society (Watts 2013). The divide between human and non-human agency reproduces and endorses colonial ways of knowing and being, and subordinates other ontologies. Deconstructing these dichotomies and re-affirming the connection between place, non-human and human is vital to access what Watts (2013) refers to as the pre-colonial mind.

The relationality between humans and the rest of nature is also a crucial way of knowing and living in many African traditional environmental knowledge systems (Abrha 2019). However, there is little attention given in the non-human agency debate to contributions of traditional environmental knowledge and African scholars. Although local and indigenous peoples develop complex relationships with both native and exotic species (Rashford 2013; Voeks 2018), native trees often relate strongly to social identities and sense of place through oral stories and symbolic roles and are therefore culturally important and likely irreplaceable. Yet, these socio-ecological complex interactions and networks are neglected in recent global environmental agendas, compromised by technocratic solutions and a lack of attention to different ways of knowing and valuing the world (Elias 2018).

### Policy implications

Any policy aimed at improving or protecting the ecosystem services provided by the néré trees or the overall landscape (e.g. a landscape restoration policy) needs to consider the social system as an integral part of the ecological system. Néré and other native trees are the essence of life itself, creating and sustaining vital social connections with fundamental meaning for the future changes the inhabitants of the West African savannah landscapes and humanity will face. The value of adding or preserving trees cannot be reduced to the value of their products, but also include how disputes are settled, and as focal point for wider social functions such as creating meaning, identity, relationships, and culture. The effect of climate- and globalisation-driven changes that affect these trees will be determined by the highly developed social system that has evolved around them. The social dynamics mediated by the tree enable many groups to negotiate and adapt their livelihoods, both directly from the tree and in other aspects of the economy. The value of the trees in enabling this social functioning needs to be considered as an integral part of the trees themselves.

Under the prospect of current global restoration agendas, with a great part of the pledges focussing on commercial tree plantations (Lewis et al. 2019), protecting indigenous trees and their mosaic landscapes is an urgent priority. In a complex and fine-tuned system of rights and benefits, any change to the ecological nodes will strongly influence social nodes by affecting the structure of the cascades of rights and benefits. Conversely, a change in a single social node also affects interactions within the ecological nodes. This entanglement between all elements of the landscape needs to be better analysed and considered to avoid negative impacts of technical interventions on the social systems and the relational web of actors. The consideration of traditional African systems as the source of wisdom and inspiration is fundamental to a transformative paradigm shift in dealing with current biodiversity and climate crisis, as suggested by many African environmentalists (Tangwa 2005; Ojomo 2011; Abrha 2019). Hence, our study of néré illustrates the remarkable untapped potential of Indigenous knowledge systems in African traditions and their theoretical values and practical relevance to inform current debates and global and national development and environmental agendas (Abrha 2019).

## Conclusion

In this paper, we applied a social–ecological network analysis approach to study the social and ecological interactions around néré, an important tree species in African savannah landscapes. We documented the myriad benefits the tree provides for diverse people, the multiple social interactions around néré and the complex network involving a large range of actors managing or benefiting from néré. Two hierarchical sub-networks represented how rights and benefits flow amongst people and highlighted power asymmetries and counter-powers. The network analysis illustrated the nuanced complexity of the interactions between social and ecological components of a social–ecological system. The findings call for thoughtfully considering the implications for global ecological agendas, such as those focused on restoration. The loss of native tree species represents more than the loss of tangible, measurable ecosystem functions and services that are assumed as restorable or replaceable by other species that provide similar functions. Such losses also entail the dissolution of fine-tuned social and environmental networks and a web of relationships and interactions reflecting diverse identities and social positions. Hence, the maintenance of indigenous culturally significant species, which have been playing the role of Cultural Keystone Species in West Africa (without being recognised yet as such), involves an awakening of a sense of place that strengthens the bonds between people, place, identities, and social and cultural diversity. This calls for prioritising protection and conservation over a substitution of species through restoration, and a radical review of how restoration ecology and practice are currently conceptualised and implemented worldwide.

## Supplementary Information

Below is the link to the electronic supplementary material.

Supplementary file 1 (PDF 1749 kb)
