# Supplementary material for: Trees as brokers in social networks: Cascades of rights and benefits from a Cultural Keystone Species
Source: Ambio. 2022 Jun 23;51(10):2137–54. doi: 10.1007/s13280-022-01733-z (PMC9378799; doi:10.1007/s13280-022-01733-z)
Supplement: Supplementary file 1 — (PDF 1749 kb) [file 13280_2022_1733_MOESM1_ESM.pdf]

Ambio

Supplementary Information

This supplementary information has not been peer reviewed.

Title: Trees as brokers in social networks for adaptation:
cascades of rights and benefits from a cultural keystone species

Authors: Houria Djoudi, Bruno Locatelli, Catherine Pehou, Matthew J. Colloff, Marléne Elias, Denis Gautier, Russell Gorddard, Barbara Vinceti, Mathurin Zida

This document provides details about the methods for building and analysing social-ecological networks to study social-ecological interactions around a tree species. The study followed six broad steps.

Step 1. Gathering data about the case study.

We conducted field work in the study site to collect qualitative and quantitative data about the social-ecological interactions around the tree. The aim was to achieve an inventory and a description of the interactions. The actors involved in Néré management, harvest, and transformation were first identified from observations and interviews and then confirmed through a household survey. The market related actors were identified through market studies.

The data used in this study came from various research projects conducted between 2013 and 2017 on land and tree tenure, value chains, and livelihoods related to Néré, in which two authors of this paper participated (Pehou et al., 2020). Our qualitative analysis was built on a synthesis of a rich dataset collected by four means.

First, ethnographic and participant observation and 36 qualitative interviews were used to gather data on the cultural, social and spiritual context and the access rights of different social groups. We observed the daily harvest activities in the fields, fallows, and woodlands. We documented land tenure and tree tenure regulations and restrictions. Through focus group discussions in 18 participatory workshops, we collected information on the actors involved in Néré activities, their relationships and exchanges, the importance of Néré for different social groups, the different types of fields and woodlands where it was harvested, the products used, the seasonality of use, and the threats to the species.

Secondly, we interviewed 180 women, randomly selected across ethnic groups: 62 Nouni, 81 Mossé, and 37 Fulani (these numbers are proportional to the population of each ethnic group across the selected villages). The semi-structured interviews dealt with the use of the Néré tree, the economic value of different Néré products, the economic and social exchanges around Néré, and the participation of household members in its management, harvest, and transformation activities.

Thirdly, we analysed the history of the sites to understand the evolution of access rights and changes in harvesting and use practices. For this, we listened to the life histories of six women aged 55 years or older and conducted semi-structured interviews with customary authorities, official authorities and technical staff from the Forest Service.

Finally, we studied the sumbala market to understand trade and use practices. For this, we surveyed 280 Néré traders and 133 consumers in 24 selling sites: traditional markets, food stores and shops around the study area and in the capital city of Ouagadougou.

Step 2. Defining nodes.

Based on our knowledge of the field and the interactions described in the data, we defined the key human and non-human elements of the social-ecological system, which would become the nodes of the social-ecological network. We identified the ecological nodes (i.e., land, animals, trees, seeds on trees, and harvested seeds) and the social nodes (i.e., actors related to Néré). The list of social nodes included local inhabitants (described by their gender, marital status, age, and ethnicity, for example migrant Mossé or first spouses and other spouses of Nouni farmers) and external actors (described by their role, such as traders or consumers). The social nodes were types of actors (e.g. pastoralists) rather than individuals (e.g., Mr X, a pastoralist).

We also included a few meta-nodes (e.g., “any community member”) to ease the coding of links. For example, as any community member may help people facing hardship, we create one interaction from “all community members” to “people in hardship”. At the time of creating the mathematical network, the “all community members” node was replaced by several nodes (one for each type of community members) and the interaction was duplicated.

We created a spreadsheet with the node list and details (Table SI1).

Table S1: Node list (S for social nodes, E for ecological nodes, G for meta-nodes)

ID	Description	Content (for meta-nodes)
S01	Native Farmer Men or Chiefs	
S02	Native Farmer First Spouses	
S03	Native Farmer Other Spouses	
S04	Native Farmer Other Family Members	
S05	Migrant Farmer Men	
S06	Migrant Farmer Youth	
S07	Migrant Farmer First Spouses	
S08	Migrant Farmer Other Spouses	
S09	Migrant Farmer Other Family Members	
S10	Pastoralist Men	
S11	Pastoralist Women	
S12	Visitors	
S13	People Facing Hardship	
S14	Rural Consumers	
S15	Female Urban Traders	
S16	Urban and Foreign Consumers	
S17	Consumers in the North	
E01	Land	
E02	Animals	
E03	Trees	
E04	Seeds on Trees	
E05	Harvested Seeds	
G01	Native Farmer Women	S02 S03
G02	Migrant Farmer Women	S07 S08
G03	All Community Members	S01 S02 S03 S04 S05 S06 S07 S08 S09
G04	Native Farmer Family Members	S01 S02 S03 S04
G05	Migrant Farmer Family Members	S05 S07 S08 S09

Step 3. Listing interactions.

We extracted information on social-ecological interactions to create a list of network links. For this, we browsed the collected data and recorded all mentioned interactions related to Néré. We excluded interactions that were mentioned only once to avoid overcomplicating the network with marginal or anecdotal links.

Step 4. Mapping the social-ecological system.

We drew the nodes on a whiteboard and mapped the interactions among them by drawing links on the board. The process was similar to a concept mapping or related approaches from systems thinking (Aubrecht et al., 2019; Davies, 2011). During this process, we defined a typology of links (Table SI2). The typology was not predefined but created from the data.

Table S2. Link types

Link type	Nodes	Link description
**Right**	Social to Social	A influences the rights of B over land or trees (e.g., granting access, authorizing harvest, restricting or controlling uses)
**Transfer**	Social to Social	A transfers to B the contributions from Néré (e.g., giving or selling seeds)
**Work**	Social to Social	A helps B to conduct an action related to land or trees (e.g., participating in harvest)
**Payment**	Social to Social	A pays B with cash or goods (goods that are not Néré products) (e.g., paying in cash to buy seeds)
**Ecology**	Ecological to Ecological	An ecological element influences another (e.g., trees providing fodder to animals)
**Action**	Social to Ecological	A conducts an action on an ecological element (e.g., harvesting or selling seeds)
**Contribution**	Ecological to Social	An ecological element provides a contribution to the livelihoods or wellbeing of A (e.g., providing shade, income or nutrition)

We built the map iteratively by adding new interactions at each iteration (see examples of initial and almost final maps in Fig. SI1 and SI2). At one point, it became clear that the drawing would be facilitated by separating the system into three levels, depending on where the interactions took place (land, tree, or product levels).

After each map was created, we checked it for inconsistencies, redundancies or omissions. Whereas the first map was drawn on a whiteboard, we drew the following ones with the VUE (Visual Understanding Environment) software (Tufts University, 2015).

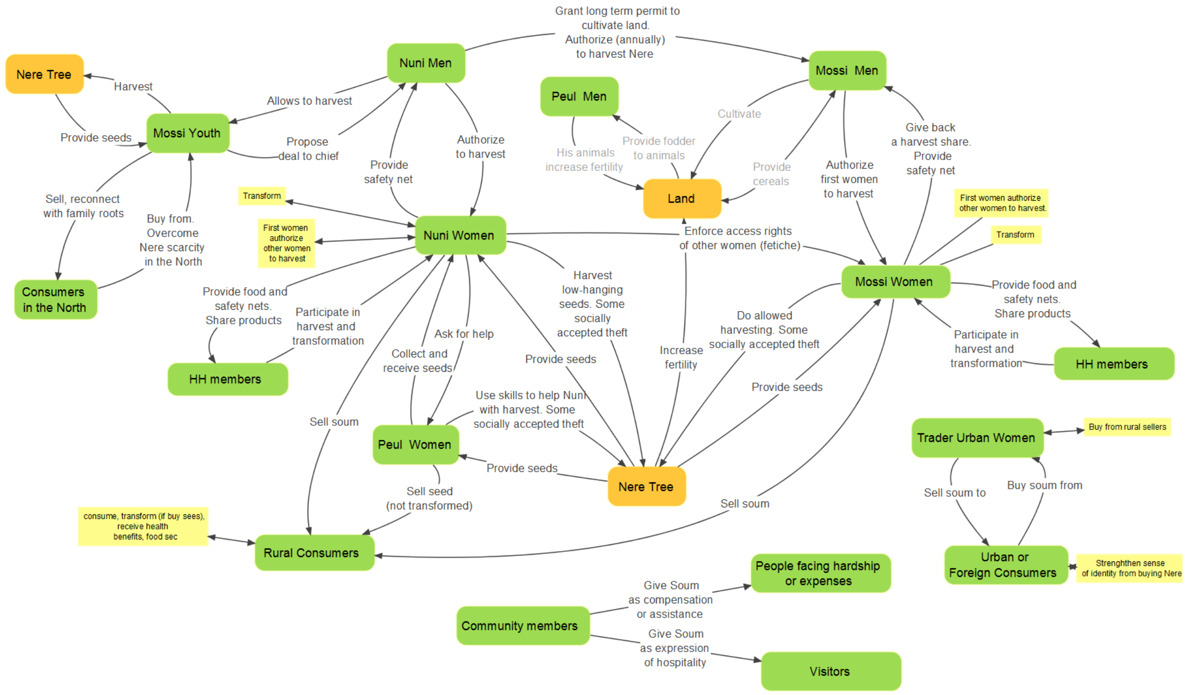

Figure S1. One of the first maps of the social-ecological interactions

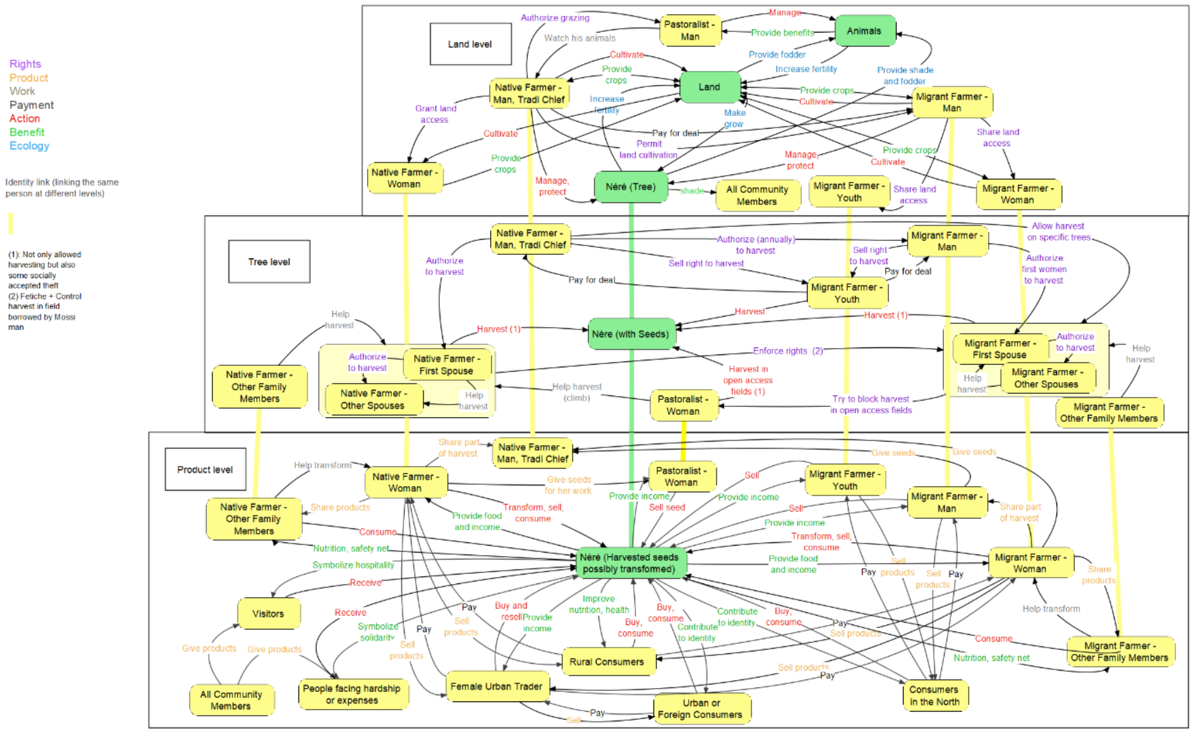

Figure S2. One of the almost final maps of the social-ecological interactions

When the final map was agreed upon by the authors, we created a spreadsheet with the list of links included in the map. The links were described by the key attributes "Type", "Source Node", "Target Node", and "Level" (Table SI3).

Step 5. Creating the mathematical network.

We imported both spreadsheets (node list and interaction list) in R to build the mathematical network. We converted the meta-nodes into the corresponding nodes and duplicated the links from or to these meta-nodes. We transformed the information on interactions into an adjacency matrix, i.e. a square asymmetrical matrix with values of 0 and 1 and rows or columns representing nodes. A value of 1 in the matrix meant that there was a link from the row node to the column node.

In order to retain the information on the types of links (e.g., Rights, Work, or Payment), we built seven adjacency matrices, one per link type. We then created seven networks from the adjacency matrices with the "graph_from_adjacency_matrix" function in the R package *igraph* (Csardi, 2018). These seven networks were combined to create a multiplex network with seven layers (one by type of interactions). In this multiplex network (Fig. SI3), all nodes can exist in all layers and the links between different nodes occur within layers. The inter-layer links connect one node in one layer to the same node in another layer and are called "self-coupling" links.

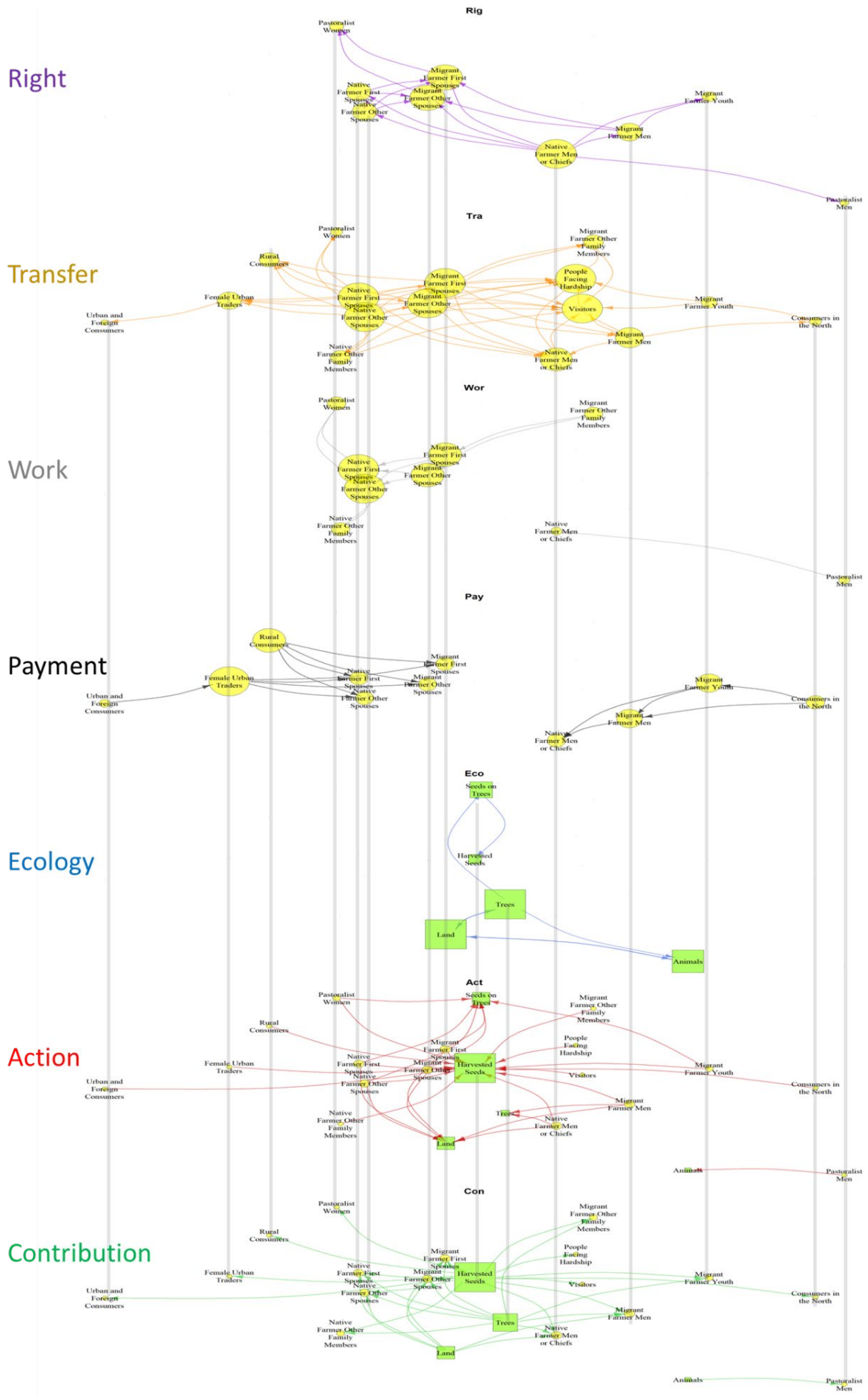

Figure S3. The multiplex network. Each layer corresponds to a type of interaction. The same node can exist in several layers. A grey vertical link connects a node in one layer with the same node in another layer (self-coupling link)

The multiplex network was created as *igraph* object with an attribute “Level” for links. We plotted the full network using a force-directed layout algorithm (Fruchterman and Reingold, 1991), in which two nodes are close to each other if they are linked (which is visually depicted as a group of connected nodes that are in the same region of the plot) (see flattened version of the multiplex network in SI4).

With the multiplex network, we described the types of observed interactions between pairs of nodes and their number of occurrences in the network. These interactions were either reciprocal (two links of opposite directions connect two nodes) or not. We also calculated the degree of each node (number of links to or from a node).

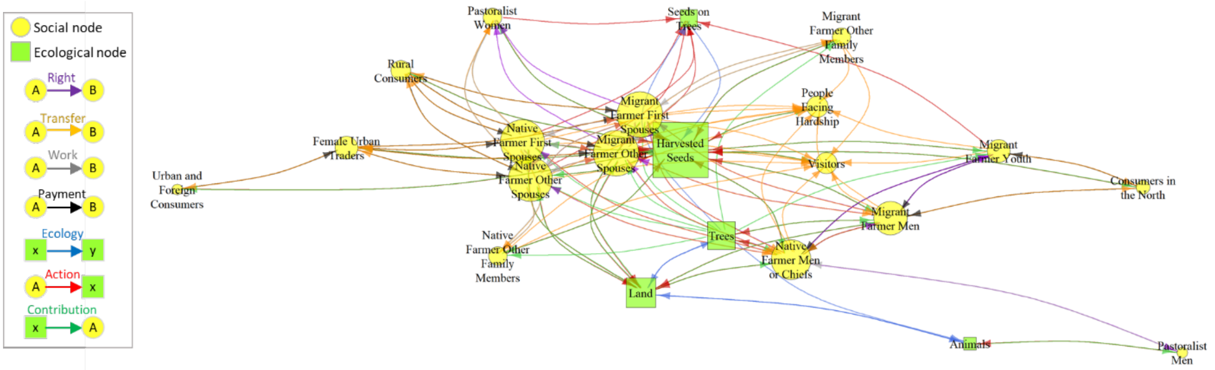

Figure S4. Flattened representation of the multiplex network (the area of each node is proportional to its degree and the node label shows the node degree d).

Step 6. Analysing sub-networks.

We focused on two sub-networks, both built using the full set of nodes and a subset of selected links. The first one, called ‘network of rights’, showed how people exchanged rights among themselves and acted on ecological elements (selected links were “Right” and “Action”). The second one, called ‘network of benefits’, showed how ecological elements interacted and produced contributions to people (e.g. supplying seeds) and how people interacted to exchange these contributions (e.g. giving seeds) (selected links were “Ecology”, “Contribution”, and “Transfer”). We drew these two sub-networks and analysed their hierarchical nature. A hierarchical network is one in which paths (sequences of links) are not reciprocated, for example, if node A influences node B, node B cannot influence node A, directly or indirectly through other nodes. Hierarchical networks show the pyramidal structure of a group, in which dominant actors can be identified.

We calculated a simple index to identify the degree of hierarchy of the full network and the two sub-networks (the Krackhardt hierarchy score, which has a value of 1 for a fully hierarchical network and 0 for a non-hierarchical one) (Krackhardt, 1994). The Krackhardt hierarchy measure was calculated with the *hierarchy* function in the *sna* package for (Butts, 2016).

In the hierarchical sub-networks, we identified the dominant actors, i.e. the nodes that connect to a

large number of other nodes following the directed links of a network. In the sub-network of rights (respectively benefits), this would be the nodes that can transfer rights (benefits) to many other nodes, directly or indirectly. The two sub-networks were plotted using a layout algorithm for hierarchical graph drawing (Sugiyama et al., 1981), in which the most dominant actors are at closest to the top (Fig. SI5). The dominance was measured as the length of subcomponents with *igraph*, and more specifically with the function “*subcomponent(myGraph , j, mode="out")*”, which determines all nodes reachable from a give node *j* via a directed path through the network *myGraph*.

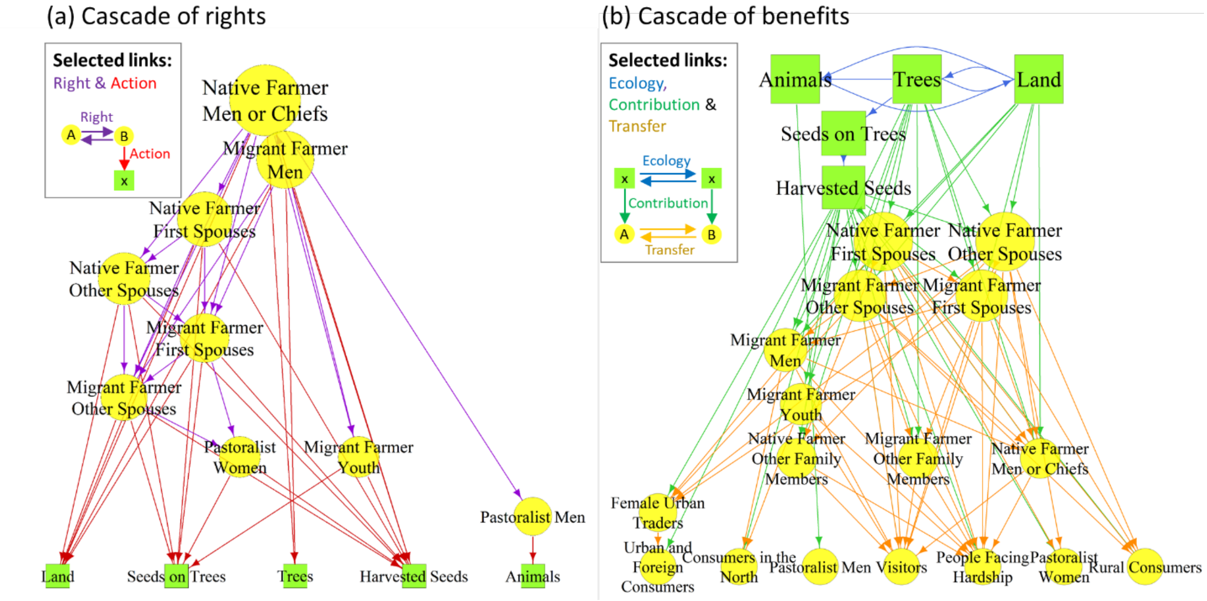

Figure S5. The two sub-networks representing a cascade of rights (a) and a cascade of benefits (b). The link selected for each sub-network are shown in the top-left box. Node size is proportional to the dominance of the actors (or the distance from the bottom of the graph).

Table S3. Interaction list

Type	Source Node	Target Node	Short description	Level
**Ecology**	Land	Animals	Provide fodder	Land
**Ecology**	Animals	Land	Increase fertility	Land
**Ecology**	Land	Trees	Make growth	Land
**Ecology**	Trees	Land	Increase fertility	Land
**Ecology**	Trees	Animals	Provide shade and fodder	Land
**Ecology**	Trees	Seeds on Trees	Make growth	Trees
**Ecology**	Seeds on Trees	Harvested Seeds	Result in	Trees
**Right**	Native Farmer Men or Chiefs	Pastoralist Men	Authorize grazing	Land
**Work**	Pastoralist Men	Native Farmer Men or Chiefs	Watch his animals	Land
**Action**	Pastoralist Men	Animals	Menage	Land
**Contribution**	Animals	Pastoralist Men	Provide benefits	Land
**Action**	Migrant Farmer Men	Land	Cultivate	Land
**Contribution**	Land	Migrant Farmer Men	Provide cereals	Land
**Action**	Migrant Farmer Women	Land	Cultivate	Land
**Contribution**	Land	Migrant Farmer Women	Provide cereals	Land
**Action**	Native Farmer Men or Chiefs	Land	Cultivate	Land
**Contribution**	Land	Native Farmer Men or Chiefs	Provide cereals	Land
**Action**	Native Farmer Women	Land	Cultivate	Land
**Contribution**	Land	Native Farmer Women	Provide cereals	Land
**Right**	Migrant Farmer Men	Migrant Farmer Youth	Share land access	Land
**Right**	Migrant Farmer Men	Migrant Farmer Women	Share land access	Land
**Right**	Native Farmer Men or Chiefs	Migrant Farmer Men	Grant long-term cultivation permit	Land
**Payment**	Migrant Farmer Men	Native Farmer Men or Chiefs	Pay for the right to cultivate (generally in nature: amount of cereals per area)	Land
**Right**	Native Farmer Men or Chiefs	Native Farmer Women	Grant land access	Land
**Action**	Native Farmer Men or Chiefs	Trees	Menage, protect	Land
**Contribution**	Trees	Native Farmer Men or Chiefs	Mark land	Land
**Action**	Migrant Farmer Men	Trees	Menage, protect	Land
**Right**	Native Farmer Men or Chiefs	Native Farmer First Spouses	Authorize to harvest	Trees
**Right**	Native Farmer First Spouses	Native Farmer Other Spouses	Authorize to harvest	Trees
**Work**	Native Farmer Other Spouses	Native Farmer First Spouses	Help harvest	Trees
**Work**	Native Farmer Other Family Members	Native Farmer Women	Help harvest	Trees
**Right**	Migrant Farmer Men	Migrant Farmer First Spouses	Authorize to harvest	Trees
**Right**	Migrant Farmer First Spouses	Migrant Farmer Other Spouses	Authorize to harvest	Trees
**Work**	Migrant Farmer Other Spouses	Migrant Farmer First Spouses	Help harvest	Trees
**Work**	Migrant Farmer Other Family Members	Migrant Farmer Women	Help harvest	Trees
**Right**	Native Farmer Men or Chiefs	Migrant Farmer Men	Authorize harvest annually	Trees
**Transfer**	Migrant Farmer Men	Native Farmer Men or Chiefs	Give back seeds to keep good relationships	Trees
**Right**	Native Farmer Men or Chiefs	Migrant Farmer Women	Authorize harvest on specific trees	Trees
**Transfer**	Migrant Farmer Women	Native Farmer Men or Chiefs	Give back seeds to keep good relationships	Trees
**Right**	Native Farmer Men or Chiefs	Migrant Farmer Youth	Sell right to harvest	Trees
**Payment**	Migrant Farmer Youth	Native Farmer Men or Chiefs	Pay for deal	Trees
**Right**	Migrant Farmer Men	Migrant Farmer Youth	Sell right to harvest	Trees
**Payment**	Migrant Farmer Youth	Migrant Farmer Men	Pay for deal	Trees
**Action**	Native Farmer Women	Seeds on Trees	Harvest	Trees
**Action**	Migrant Farmer Women	Seeds on Trees	Harvest	Trees

**Action**	Migrant Farmer Youth	Seeds on Trees	Harvest	Trees
**Action**	Pastoralist Women	Seeds on Trees	Harvest in open access fields	Trees
**Right**	Migrant Farmer Women	Pastoralist Women	Try to block harvest in open access fields	Trees
**Right**	Native Farmer Women	Migrant Farmer Women	Enforce rights	Trees
**Work**	Pastoralist Women	Native Farmer Women	Help harvest (climb)	Trees
**Transfer**	Native Farmer Women	Pastoralist Women	Give seeds for her work	Seeds
**Work**	Migrant Farmer Women	Native Farmer Women	Help harvest	Trees
**Transfer**	Native Farmer Women	Migrant Farmer Women	Give seeds for her work	Seeds
**Work**	Native Farmer Other Family Members	Native Farmer Women	Help transform	Seeds
**Transfer**	Native Farmer Women	Native Farmer Other Family Members	Share Transfers	Seeds
**Work**	Migrant Farmer Other Family Members	Migrant Farmer Women	Help transform	Seeds
**Transfer**	Migrant Farmer Women	Migrant Farmer Other Family Members	Share Transfers	Seeds
**Transfer**	Native Farmer Women	Native Farmer Men or Chiefs	Share part of harvest	Seeds
**Transfer**	Migrant Farmer Women	Migrant Farmer Men	Share part of harvest	Seeds
**Action**	Native Farmer Women	Harvested Seeds	Transform, sell, consume	Seeds
**Contribution**	Harvested Seeds	Native Farmer Women	Provide food and income	Seeds
**Action**	Migrant Farmer Women	Harvested Seeds	Transform, sell, consume	Seeds
**Contribution**	Harvested Seeds	Migrant Farmer Women	Provide food and income	Seeds
**Action**	Pastoralist Women	Harvested Seeds	Sell seeds	Seeds
**Contribution**	Harvested Seeds	Pastoralist Women	Provide income	Seeds
**Action**	Migrant Farmer Youth	Harvested Seeds	Sell	Seeds
**Contribution**	Harvested Seeds	Migrant Farmer Youth	Provide income	Seeds
**Action**	Migrant Farmer Men	Harvested Seeds	Sell	Seeds
**Contribution**	Harvested Seeds	Migrant Farmer Men	Provide income	Seeds
**Transfer**	Migrant Farmer Youth	Consumers in the North	Sell Transfers	Seeds
**Payment**	Consumers in the North	Migrant Farmer Youth	Pay	Seeds
**Transfer**	Migrant Farmer Men	Consumers in the North	Sell Transfers	Seeds
**Payment**	Consumers in the North	Migrant Farmer Men	Pay	Seeds
**Action**	Native Farmer Family Members	Harvested Seeds	Consume	Seeds
**Contribution**	Harvested Seeds	Native Farmer Family Members	Nutrition, safety net	Seeds
**Action**	Migrant Farmer Family Members	Harvested Seeds	Consume	Seeds
**Contribution**	Harvested Seeds	Migrant Farmer Family Members	Nutrition, safety net	Seeds
**Action**	Female Urban Traders	Harvested Seeds	Buy and sell	Seeds
**Contribution**	Harvested Seeds	Female Urban Traders	Provide income	Seeds
**Action**	Rural Consumers	Harvested Seeds	Buy, consume	Seeds
**Contribution**	Harvested Seeds	Rural Consumers	Improve nutrition, health	Seeds
**Action**	Urban and Foreign Consumers	Harvested Seeds	Buy, consume	Seeds
**Contribution**	Harvested Seeds	Urban and Foreign Consumers	Contribute to identity	Seeds
**Action**	Consumers in the North	Harvested Seeds	Buy, consume	Seeds
**Contribution**	Harvested Seeds	Consumers in the North	Contribute to identity	Seeds
**Transfer**	All Community Members	People Facing Hardship	Give Transfers	Seeds
**Transfer**	All Community Members	Visitors	Give Transfers	Seeds
**Action**	People Facing Hardship	Harvested Seeds	Receive	Seeds
**Contribution**	Harvested Seeds	People Facing Hardship	Symbolize solidarity	Seeds
**Action**	Visitors	Harvested Seeds	Receive	Seeds
**Contribution**	Harvested Seeds	Visitors	Symbolize hospitality	Seeds
**Transfer**	Native Farmer Women	Female Urban Traders	Sell Transfers	Seeds
**Payment**	Female Urban Traders	Native Farmer Women	Pay	Seeds
**Transfer**	Native Farmer Women	Rural Consumers	Sell Transfers	Seeds
**Payment**	Rural Consumers	Native Farmer Women	Pay	Seeds
**Transfer**	Migrant Farmer Women	Female Urban Traders	Sell Transfers	Seeds
**Payment**	Female Urban Traders	Migrant Farmer Women	Pay	Seeds
**Transfer**	Migrant Farmer Women	Rural Consumers	Sell Transfers	Seeds
**Payment**	Rural Consumers	Migrant Farmer Women	Pay	Seeds
**Transfer**	Female Urban Traders	Urban and Foreign Consumers	Sell Transfers	Seeds
**Payment**	Urban and Foreign Consumers	Female Urban Traders	Pay	Seeds
**Contribution**	Trees	All Community Members	Provide shade	Land

References

Aubrecht, K.B., Dori, Y.J., Holme, T.A., Lavi, R., Matlin, S.A., Orgill, M., Skaza-Acosta, H. (2019) Graphical Tools for Conceptualizing Systems Thinking in Chemistry Education. Journal of Chemical Education 96, 2888-2900.

Butts, C.T., (2016) sna: Tools for Social Network Analysis. R package version 2.4. https://CRAN.R-project.org/package=sna.

Csardi, G., (2018) igraph: Routines for simple graphs and network analysis, version 1.2.2. https://cran.r-project.org/package=igraph.

Davies, M. (2011) Concept mapping, mind mapping and argument mapping: what are the differences and do they matter? Higher education 62, 279-301.

Fruchterman, T.M., Reingold, E.M. (1991) Graph drawing by force-directed placement. Software: Practice and experience 21, 1129-1164.

Krackhardt, D. (1994) Graph theoretical dimensions of informal organizations. Computational organization theory 89, 123-140.

Pehou, C., Djoudi, H., Vinceti, B., Elias, M. (2020) Intersecting and dynamic gender rights to néré, a food tree species in Burkina Faso. Journal of Rural Studies 76, 230-239.

Tufts University, (2015) Visual Understanding Environment VUE, version 3.3.0 https://vue.tufts.edu/. UIT Academic Technology, Medford, Massachusetts.
